# Genome-wide identification and expression profile of Elovl genes in threadfin fish *Eleutheronema*

**DOI:** 10.1038/s41598-023-28342-4

**Published:** 2023-01-19

**Authors:** Jie Xiao, Wen-Xiong Wang

**Affiliations:** 1 School of Energy and Environment and State Key Laboratory of Marine Pollution, City University of Hong Kong, Kowloon, Hong Kong China; 2 Research Centre for the Oceans and Human Health, City University of Hong Kong Shenzhen Research Institute, Shenzhen, 518057 China

**Keywords:** Ecology, Evolution

## Abstract

Long-chain polyunsaturated fatty acids (LC-PUFA), including eicosapentaenoic acid and docosahexaenoic acid, are the essential fatty acids for organs to maintain various biological functions and processes. The threadfin fish *Eleutheronema*, with its rich nutritional value especially the high fatty acid contents, has become one of the promising aquaculture species in China and the potential food source of fatty acids for human consumption. However, the molecular basis underlying the biosynthesis of fatty acids in *Eleutheronema* species is still unknown. The elongation of the very long-chain fatty acids (Elovl) gene family in fish plays several critical roles in LC-PUFA synthesis. Therefore, in the present study, we performed genome-wide identification of the Elovl gene family to study their evolutionary relationships and expression profiles in two threadfin fish species *Eleutheronema tetradactylum* and *Eleutheronema rhadinum*, the first representatives from the family *Eleutheronema*. Phylogenetic analysis revealed that the Elovl genes in *Eleutheronema* were classified into six subfamilies (*elovl1a/1b*, *elovl4a/4b*, *elovl5*, *elovl6/6 l*, *elovl7a*, *elovl8b*). Phylogenetic, gene structure, motif, and conserved domain analysis indicated that the Elovl genes were highly conserved within the same subfamily in *Eleutheronema*. In addition, the Elovl genes were distributed in 7/26 chromosomes, while the duplicated gene pair, *elovl4a* and *elovl4b*, showed collinear relationships. The predicted secondary structure patterns and the 3D models revealed the highly similar functions and evolutionary conserved structure of Elovl proteins in *Eleutheronema*. The selection pressure analysis revealed that Elovl genes underwent strong purifying selection during evolution, suggesting that their functions might be evolutionarily conserved in *Eleutheronema*. Additionally, the expression patterns of Elovl genes in different tissues and species were distinct, indicating the possible functional divergence during evolution in the *Eleutheronema* genus. Collectively, we provided the first comprehensive genomic information on Elovl genes in threadfin fish *Eleutheronema*. This study enhanced the understanding of the underlying mechanisms of fatty acids biosynthesis in *Eleutheronema*, and provided new insights on breeding new varieties of fatty acids-enriched fish with potential benefits to farmers and the health of consumers.

## Introduction

Fatty acids, the natural components of fats and oils, can be divided into saturated, monounsaturated, and polyunsaturated fatty acids of three classes based on their chemical structure. Saturated fatty acids are mainly found in animal foods such as meat. Unsaturated fatty acids, especially the omega-3 long-chain polyunsaturated fatty acids including eicosapentaenoic acid (EPA) and docosahexaenoic acid (DHA), are almost originated from fatty marine organisms^1^. However, polyunsaturated fatty acids cannot be synthesized by humans, and must be obtained from diet or supplementation^2^. In vertebrates, dietary long-chain polyunsaturated fatty acids (LC-PUFA) are crucial in maintaining the various biological functions and processes, including immune function, reproduction, growth, and development^3^. Moreover, fish are the primary source of LC-PUFA, including eicosapentaenoic acid (EPA) and docosahexaenoic acid (DHA), for human consumption^4^.

In vertebrates, middle chain length of saturated fatty acids, mostly C16–18, are produced by fatty acid synthase, and then desaturated and elongated into long chain fatty acids (LC-FAs) and very long chain fatty acids (VLC-FAs, chain lengths > 24 carbons). The elongation process is catalyzed by fatty acyl elongases, the Elongation of Very Long-chain fatty acids (Elovl) proteins, an essential family of membrane-bound enzymes of the elongation pathway of fatty acids^5^. Seven Elovl gene family members in mammals, including ELOVL 1–7, were successfully characterized, exhibiting dramatically different expression patterns in different tissues^6^. Generally, *elovl1*, *elovl3*, *elovl6,* and *elovl7* can elongate saturated fatty acids (SFA) and monounsaturated fatty acids (MUFA), while the preferred substrates of *elovl2*, *elovl4,* and *elovl5* were PUFA^7^. In teleost, Agabaet et al.^8^ firstly reported the Elovl gene, *elovol5*, in zebrafish. Since then, many studies have reported the molecular and functional characterization of Elovl genes from a large variety of fish species.

Due to the teleosts-specific whole-genome duplication events, the sub-functionalized paralogues of some genes have been successfully identified in various fish species^9^. Zebrafish (*D. rerio*) possess two *elovl1* members, *elovl1a* and *elovl1b*, which were required for kidney and swim bladder development during zebrafish embryogenesis^10^. The paralogues of the *elovl1* gene were also characterized in *Gymnocypris przewalskii* and played essential roles in adaptation to cold temperature^11^. The *elovl2* was essential for the biosynthesis of docosahexaenoic acid (DHA)^12^, which has been identified in various fish species, including *Lepisosteus oculatus* (NCBI accession: XP_015210453.1), *Danio rerio* (NP_001035452.1), and *Salmo salar* (NP_001130025.1). In addition, a previous study reported that the *elovl2* activity was specific to C_20_ and C_22_ PUFAs^13^. However, the loss of the *elovl2* gene as the consequence of the expansion of teleosts during evolution emerged in most marine species^6^. A recent study revealed that the compensation system in many marine teleosts for the absence of *elovl2* might be related to the gene duplication of *elovl4* (*elovl4a* and *elovl4b*), which was generally presented in most teleosts^14^. The *elovl4* has been widely identified and investigated in marine fish, responsible for the elongation toward VLC-PUFA. Most importantly, beyond their role in VLC-PUFA biosynthesis, the *elovl4* played a critical role in the biosynthesis of LC-PUFA such as DHA in organism^15^. The *elovl5* was present in virtually all teleosts that were specific for the elongation of C_18_ and C_20_ PUFAs, and also had capacity towards C_22_ PUFA in some fish, such as *Pegusa lascaris*^16^ and *Scatophagus argus*^17^. The *elovl6* acted to convert C_16_ saturated and monounsaturated fatty acids to C_18_ fatty acids, similar to mammals, which has been reported in *Larimichthys crocea*^18^ and *Oncorhynchus mykiss*^19^. The novel elongase, *elovl8*, including *elovl8a* and *elovl8b*, were apparently absent in mammals but shown to have elongation activity to C_18_ and C_20_ PUFA in teleosts such as *Clarias gariepinus* and *Siganus canaliculatus*^20^.

The threadfin *Eleutheronema* is an important marine fishery resource in Indo-Pacific regions, and has become an important and economic aquaculture species in China with high auction prices in local fish markets^21, 22^. Moreover, the *Eleutheronema* fishes were considered as a promising aquaculture species in China with a worldwide market demand because of its good flavor and high nutritional value, especially the high fatty acid contents, which could be the potential main food source of fatty acids for human consumption. Thus, the exploration of the underlying molecular basis of fatty acids biosynthesis in *Eleutheronema* species is of great importance. The Elovl gene family, a set of crucial enzymes involved in LC-PUFA biosynthesis, has been extensively investigated in fish species in recent years. However, the evolution and function of Elovl genes in *Eleutheronema* species are unknown. Therefore, in the present study, we performed a genome-wide identification of Elovl genes in *E. tetradactylum* and *E. rhadinum*, characterized their gene and protein structures, chromosomal distributions, conserved motifs, evolutionary relationships, selection pressure, and expression profiles across different tissues. To our knowledge, this is the first study on the genome-wide identification, evolutionary relationship, and gene expression analysis of gene family in the *Eleutheronema* genus, which could provide valuable genomic resources for the future functional analysis of Elovl genes in marine fishes.

## Materials and methods

### Identification of Elovl gene family from E. tetradactylum and E. rhadinum

To identify the Elovl genes in *Eleutheronema* fish, we used the elovl amino acid sequences from zebrafish as a reference and the ELO HMM model in Pfam to detect the Elovl genes in the *E. tetradactylum* and *E. rhadinum* genome sequences. The whole genome assembly of the *Eleutheronema* species yielded a total genome length of 582 Mb (N50 = 18.26 Mb) in *E. tetradactylum* and 591 Mb (N50 = 22.59 Mb) in *E. rhadinum* (unpublished data). BLASTP program were used to identify the *elovl* like genes in *E. tetradactylum* and *E. rhadinum* protein database with the e-value of e-10 as the criterion. The hidden Markov model (HMM) files corresponding to the ELO (PF01151) domains were downloaded from the Pfam protein family database and then subjected to the *E. tetradactylum* and *E. rhadinum* protein database, respectively. The ExPASy (https://web.expasy.org/protparam/) was used to compute various physical and chemical parameters of ELOVL members. SignalP-3.0 (https://services.healthtech.dtu.dk/service.php?SignalP-3.0) and TMHMM-2.0 (https://services.healthtech.dtu.dk/service.php?TMHMM-2.0) were used to predict the presence and location of signal peptide cleavage sites and transmembrane helices in Elovl proteins. The CELLO v.2.5 (http://cello.life.nctu.edu.tw/) was utilized to analyze the subcellular localization of ELOVL members. Gene structures of ELOVL members were analyzed by the Gene Structure Display Server (GSDS, http://gsds.gao-lab.org/). The conserved motifs were identified using the MEME tool (https://meme-suite.org/meme/tools/meme) with the default parameters. All the Elovl gene sequences were subjected to the MCScanX program^23^ for the chromosomal locations and collinearity analysis and then visualized using Circos software^24^. Protein–protein interaction analysis was performed on the STRING database (http://string-db.org) using Elovl genes as the queries and the zebrafish proteins as a reference. The secondary structures were eventually predicted via the SOPMA program^25^ and Vadar server^26^. The three-dimensional structures were predicted through Protein Homology/analogy Recognition Engine V 2.0 (Phyre2) server^27^. The validation of predicted protein models was assessed through Ramachandran Plot Analysis^26^. The non-synonymous substitution (Ka) and synonymous substitution (Ks) values were estimated for Elovl genes using KaKs_Calculator software^28^, and the selection mode of each gene pair was evaluated according to the Ka/Ks ratio.

### Phylogenetic analysis of Elovl genes

To investigate the evolutionary relationships of Elovl genes in the *Eleutheronema* genus, the sequences of Elovl genes from *E. tetradactylum* and *E. rhadinum* were subjected to phylogenetic analysis and compared with the 106 publicly available Elovl genes of other organisms, including *Homo sapiens*, *Mus musculus*, *Danio rerio*, *Oreochromis niloticus*, *Oncorhynchus mykiss*, *Oryzias latipes*, *Takifugu rubripes*, *Gadus morhua*, *Sander lucioperca*, *Salmo salar*, *Cyprinus carpio*, *Lepisosteus oculatus, Ictalurus punctatus*, *Tachysurus fulvidraco*, *Coregonus clupeaformis*, and *Ictalurus punctatus*. All reference sequences were aligned by the ClustalW program in MEGAX with the default parameter^29^, and the phylogenetic tree was constructed using the maximum like-hood method with 1000 replicates. The optimal protein substitution model for evolution of Elovl genes was the JTT model with gamma distribution, which was chosen based on the results using the option provided in MEGAX.

### Expression analysis of Elovl genes across different tissues

We aimed to determine the expression patterns of Elovl genes across different tissues. In brief, adult *E. tetradactylum* and *E. rhadinum* were collected from China's coastal water. This study was reported in accordance with ARRIVE guidelines. Total RNAs of 9 different tissues, including the brain, eye, gill, heart, kidney, liver, muscle, stomach, and intestine, were isolated using RNA Extraction Kit (Takara, Japan) following the manufacturer's procedure. The RNA integrity was detected by the RNA Nano 6000 Assay Kit of the Agilent Bioanalyzer 2100 system (Agilent Technologies, CA, USA) and 1% agarose gels. The extracted RNAs (2 μg) of each tissue (*n* = 3 replicates) were respectively reverse transcribed into cDNA using the PrimeScript™ RT reagent Kit (Takara, Japan). The primers used for qPCR analysis in this study are shown in Table 1. The total qPCR mixture volume of 20 μL contained 10 μL of 2 × SYBR Green PCR buffer, 0.4 μL of forward and reverse primers, 1 μL of cDNA template, and 7.4 μL ddH_2_O. The qPCR analysis was performed in triplicates on a 96-well rotor in 7300 RT-PCR system (Applied Biosystems, USA) with the following amplification program: predenaturation at 95 °C for 30 s; denaturation at 95 °C for 5 s, annealing at 58 °C for 30 s, 40 cycles. The data were analyzed statistically using the 2^−ΔΔCt^ method using the *β-actin* as the internal reference gene.

**Table 1:** Gene-specific primers for qPCR.

Species	Gene name	Forward primer	Reverse primer
*E. tetradactylum*	*elovl1a*	ATGGTGAATGCAGGAGTCCA	TGTAAGCTGGATGGCAGTCA
	*elovl1b*	TGGATGTACGGCACCTTCTT	GATGCCGTTGCCATTCTCAT
	*elovl4a*	GGGCTGCAAGCTACAGTTAC	GAACATGGTGCAATGGTGGT
	*elovl4b*	GCAACCATCAACTCTGGCAT	ATGGTCACGTGGAACTGGAT
	*elovl5*	CATATGGCCGTGTGACTTCC	AAGAGAGCCATTCTGGTGCT
	*elovl6*	GTCCATCCCACATGCAGAAC	ACTCGCTCTTCTTGGTGACA
	*elovl6l*	GTCATTTGCCTTCGTCGTGA	AGCACAGTGATGTGGTGGTA
	*elovl7a*	GGGCCTCGGATAATGGAGAA	ATGGCCTGTGGTGAATCAGA
	*elovl8b*	GGCACCATGATCTTCAACTG	CACTCTGTGAACAAGTTGTAGC
	*β-actin*	CTGGACTTCGAGCAGGAGAT	GGATTCCGCAGGACTCCATA
*E. rhadinum*	*elovl1a*	ATGGTGAATGCAGGAGTCCA	TGTAAGCTGGATGGCAGTCA
	*elovl1b*	CCCTGGTGGAATGGGATCTT	ACCAGGACGAACTGGGTAAG
	*elovl4a*	GGGCTGCAAGCTACAGTTAC	GAACATGGTGCAATGGTGGT
	*elovl4b*	GCAACCATCAACTCTGGCAT	ATGGTCACGTGGAACTGGAT
	*elovl5*	CATATGGCCGTGTGACTTCC	AAGAGAGCCATTCTGGTGCT
	*elovl6*	GTCCATCCCACATGCAGAAC	ACTCGCTCTTCTTGGTGACA
	*elovl6l*	GTCATTTGCCTTCGTCGTGA	AGCACAGTGATGTGGTGGTA
	*elovl7a*	GGGCCTCGGATAATGGAGAA	ATGGCCTGTGGTGAATCAGA
	*elovl8b*	CCACATCAGGTGGAATGCTG	CCACATCTGGTTCACCTCCT
	*β-actin*	CTGGACTTCGAGCAGGAGAT	GGATTCCGCAGGACTCCATA

### Ethics declarations

All experimental protocols were approved by the Research Committee of City University of Hong Kong. All methods were carried out in accordance with the relevant guidelines and regulations of the City University of Hong Kong.

## Results and discussion

### Identification of Elovl genes from E. tetradactylum and E. rhadinum

Totally, we successfully identified 9 Elovl genes, including *elovl1a*, *elovl1b*, *elovl4a*, *elovl4b*, *elovl5*, *elovl6*, *elovl6l*, *elovl7a*, and *elovl8b*, both from *E. tetradactylum* and *E. rhadinum* genome (Table 2). In *E. rhadinum*, the shortest and the longest putative CDS length among all Elovl genes was 810 bp and 2019 bp, respectively. Their encoded protein size ranged from 269 amino acids to 672 amino acids. The theoretical molecular weight of Elovl proteins varied from 31061.48 to 75051.42 Da, with the theoretical isoelectric points (pI) ranging from 7.86 to 9.59. Most of the Elovl proteins were characterized as stable and hydrophilic proteins. Signal peptide prediction analysis showed that the *elovl1b*, *elovl5*, and *elovl6* contained signal peptide sequences. In addition to elovl8b, all Elovl proteins contained transmembrane domains ranging from 5 to 7. Almost all Elovl proteins were predicted to be endoplasmic reticulum-located except *elovl8b*, predominantly localized in the nucleus.

**Table 2:** Basic information for the Elovl gene family members.

Species	Gene name	putative CDS (bp)	Size (aa)	Molecular weight (Da)	pI	Instability index	Aliphatic index	Hydropathicity	Transmembrane domain	Signal peptide	Subcellular localization
*E. rhadinum*	*elovl1a*	942	313	37,022.53	9.58	36.07	88.69	0.164	7	NO	Endoplasmic reticulum
	*elovl1b*	981	326	37,938.41	9.32	29.79	85.86	0.13	7	YES	Endoplasmic reticulum
	*elovl4a*	1077	358	41,720.58	9.6	37.67	83.1	− 0.1	6	NO	Endoplasmic reticulum
	*elovl4b*	963	320	37,086.3	9.59	32.31	85.94	0.021	6	NO	Endoplasmic reticulum
	*elovl5*	885	294	35,197.1	9.11	36.69	86.84	0.041	7	YES	Endoplasmic reticulum
	*elovl6*	810	269	31,560.24	9.38	46.26	89.55	0.234	5	YES	Endoplasmic reticulum
	*elovl6l*	825	274	31,061.48	8.72	50.98	94.67	0.368	5	NO	Endoplasmic reticulum
	*elovl7a*	885	294	34,564.52	9.14	28.66	86.5	0.241	7	NO	Endoplasmic reticulum
	*elovl8b*	2019	672	75,051.42	7.86	42.73	92.41	− 0.311	0	NO	Nucleus
*E. tetradactylum*	*elovl1a*	942	313	36,886.42	9.63	34.02	89.94	0.178	7	YES	Endoplasmic reticulum
	*elovl1b*	981	326	37,928.33	9.32	30.38	82.88	0.103	7	NO	Endoplasmic reticulum
	*elovl4a*	1077	358	41,763.61	9.64	37.67	82.01	− 0.123	6	NO	Endoplasmic reticulum
	*elovl4b*	975	324	37,605.01	9.57	33.4	88.77	0.064	7	NO	Endoplasmic reticulum
	*elovl5*	885	294	35,197.1	9.11	36.69	86.84	0.041	7	YES	Endoplasmic reticulum
	*elovl6*	810	269	31,560.24	9.38	46.26	89.55	0.234	5	YES	Endoplasmic reticulum
	*elovl6l*	825	274	31,049.42	8.72	50.36	93.25	0.342	5	NO	Endoplasmic reticulum
	*elovl7a*	885	294	34,534.49	9.14	29.08	86.84	0.252	7	NO	Endoplasmic reticulum
	*elovl8b*	1824	607	68,750.14	8.93	46.33	83.48	− 0.264	3	NO	Nucleus

In *E. tetradactylum*, the putative CDS length of Elovl genes ranged from 810 to 1824 bp, and their encoded protein size ranged from 269 amino acids to 409 amino acids. The molecular weight of Elovl proteins varied from 31049.42 to 68750.14 Da, with the pI ranging from 8.72 to 9.64. Like Elovl proteins in *E. rhadinum*, most elovl proteins were predicted to be stable and hydrophilic. Signal peptide prediction analysis revealed that *elovl1a*, *elovl5*, and *elovl6* had signal peptide sequence, which was different from *E. rhadinum* that elovl1b contained signal peptide sequence, but elovl1a did not. In addition, seven members showed the same number of transmembrane structures with *E. rhadinum*, while the *elovl8b* contained three and elovl4b contained seven transmembrane structures in contrast to *E. rhadinum*. The *elovl8b* was predicted to be localized in nuclear, while other members were localized in the endoplasmic reticulum, similar to *E. rhadinum*.

### Evolution of divergence and conservation of Elovl genes

**Figure 1:**
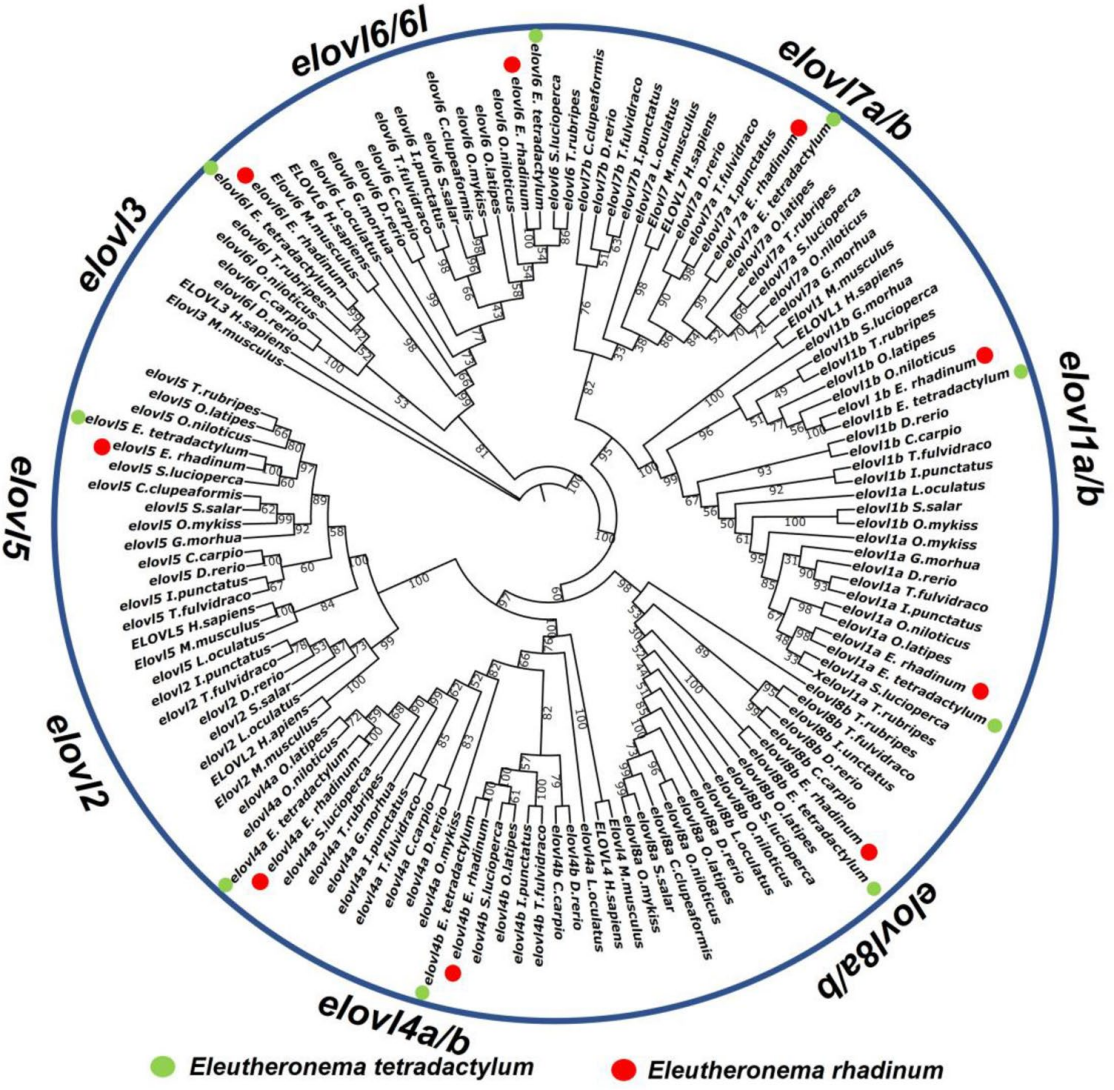
Phylogenetic tree for 18 Elovl proteins from *E. tetradactylum* and *E. rhadinum*, and 106 publicly available Elovl proteins from other species. All these proteins were aligned using ClustalW and then subjected to MEGAX for phylogenetic tree construction using the maximum like-hood method with 1000 replicates.

Divergence and conservation accompany the process of species evolution. To elucidate the phylogenetic relationship of Elovl genes among different species, a maximum like-hood tree was constructed on the basis of 18 Elovl genes in *E. tetradactylum* and *E. rhadinum* and 106 publicly available Elovl protein sequences. As shown in Fig. 1, these Elovl genes can be divided into eight subfamilies, including *elovl1a/1b*, *elovl2*, *elovl3*, *elovl4a*, *elovl5*, *elovl6/6 l*, *elovl7a/7b*, *elovl8a/8b*. However, 6 subfamilies were presented in the *Eleutheronema* genus, and there was only one subtype for *elovl7* (*elovl7a*) and *elovl8* (*elovl8b*) in *E. tetradactylum* and *E. rhadinum*. The *elovl3* was mainly identified in mammalians such as *Homo sapiens* and *Mus musculus*, while a recent study reported a full repertoire of Elovl genes in the *Colossoma macropomum* genome, including *elovl3*^30^. The loss of *elovl2* occurred in the vast majority of marine fish lineages, which was only presented in a few fish species, such as *C. carpio*, *D. rerio*, *S. salar*, and *S. grahami*.

**Figure 2:**
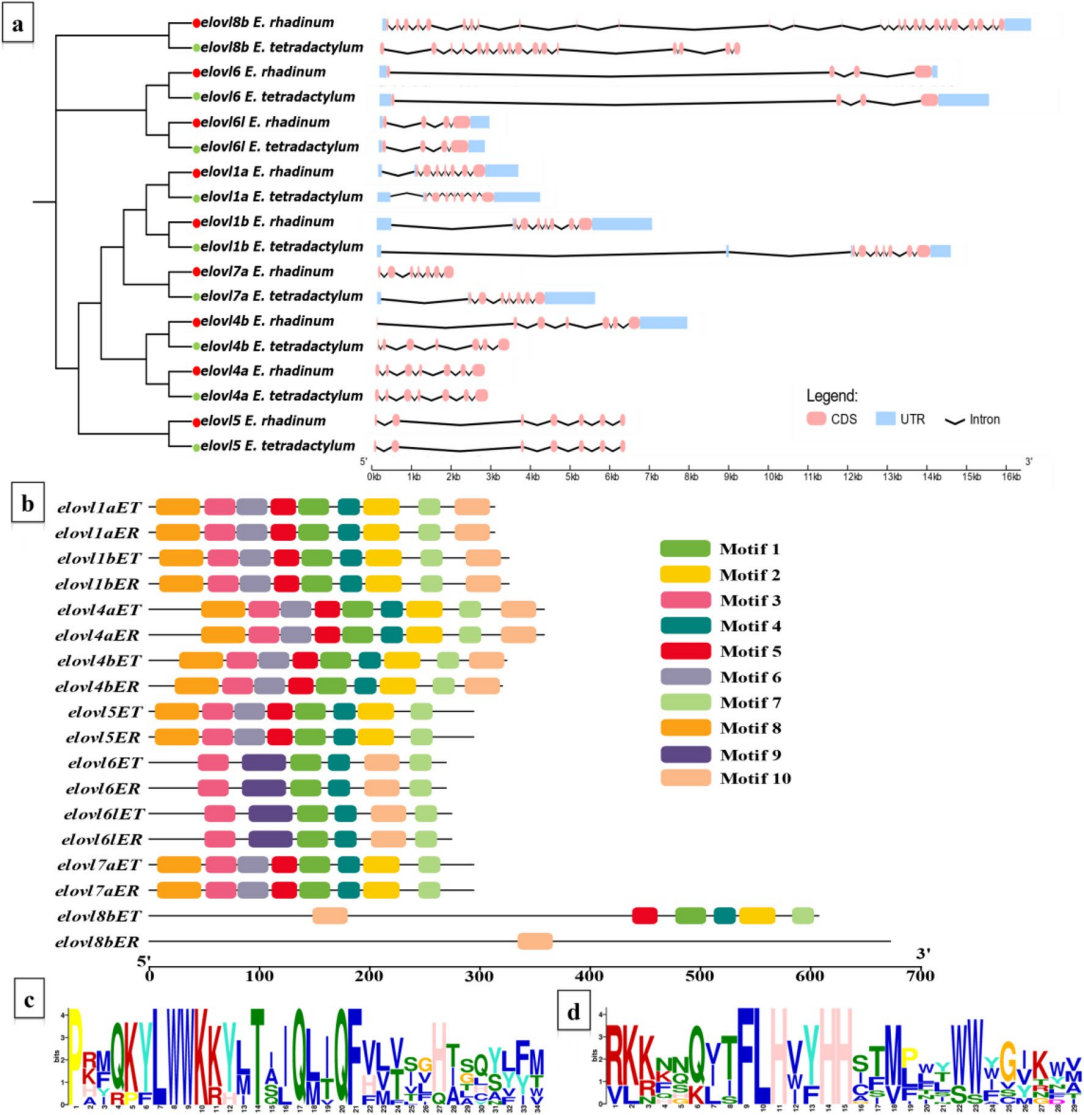
Gene structure and conserved motifs diagram of Elovl genes. (**a**) Gene structure of Elovl genes. Exons were represented by pink boxes and introns by black lines; (**b**) Conserved motifs of Elovl proteins; (**c** and **d**) Logo representations of the ELO domains, motifs 1 and 2, respectively.

We further performed the gene structure analysis to visualize the exon–intron structure of each gene, and the results revealed that the *elovl8b* had the largest intron number, while the *elovl6/6 l* subfamily genes contained three introns (Fig. 2a). Except for *elovl8*, Elovl genes belonging to the same subfamily shared a similar gene structure. Additionally, we identified ten motifs in Elovl genes, and the conversed motif types, numbers, and distributions in Elovl proteins were much more similar except for the *elovl8b* (Fig. 2b, TableS1). Two conserved motifs were found in the Elovl gene family except for *elovl8b* in *E. rhadinum*, which were related to the ELO domain via SMART evaluation analysis (Fig. 2c and d). Gene structural variation is important for gene evolution. In *E. tetradactylum* and *E. rhadinum*, Elovl genes showed similar gene structure, and the proteins shared similar motif compositions, indicating that the Elovl genes were highly conserved in the *Eleutheronema* genus.

In the process of evolution via natural selection, adaptation to certain environmental conditions likely drove the changes in endogenous capacity for LC-PUFA biosynthesis between marine and freshwater fishes^31^. The Elovl gene family has been functionally studied and characterized in a variety of fish species, and the member of the Elovl gene family of each species varied greatly. In the present study, for a comprehensive analysis of Elovl genes in the *Eleutheronema* genus, the Elovl gene ortholog clusters of mammals and various teleosts with different ecological niches and habitats were collected. The results showed that only seven Elovl genes (one gene for each subtype) were observed in mammals; however, more members were variably presented in teleosts, which might be related to the teleost-specific duplication. A previous study revealed that *Sinocyclocheilus graham* and *C. carpio* possessed the highest number of Elovl genes, containing 21 members of subtypes, resulting from an extra independent 4th whole-genome duplication event^32, 33^. Interestingly, only 9 Elovl genes were observed in *Eleutheronema* genus, the same as *T. rubripes*, possibly due to gene loss and the asymmetric acceleration of the evolutionary rate in one of the paralogs following the whole-genome duplication in some teleost fishes^34^. Additionally, the *elovl2* and *elovl3* were absent, but a novel subtype, *elovl8*, was present in most marine fishes. The *elovl8*, the most recently identified and novel active member of the Elovl protein family member, has been proposed to be a fish-specific elongase with two gene paralogs (*elovl8a* and *elovl8b*) described in teleost^35^. In *Eleutheronema*, we also found that the *elovl8b* was presented in *E. tetradactylum* and *E. rhadinum*, indicating the important roles in the LC-PUFAs biosynthesis of *Eleutheronema* fish. Similar results were also observed in rabbitfish and zebrafish^20^. The Elovl gene family member number in *Eleutheronema* genus is the same as *T. rubripes*, but less than *I. punctatus* (10), *Gadus morhua* (10), *D. rerio* (14), *S. salar* (18), and *C. carpio* (21), which might be due to the differential expansion events during the evolutions of fish species.

**Figure 3:**
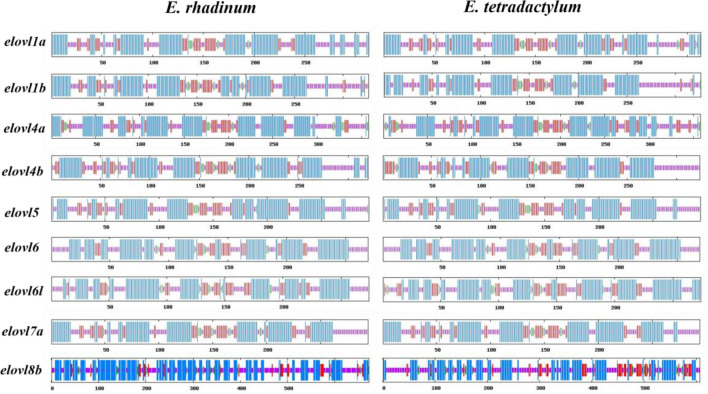
The secondary structure pattern, including alpha helix (blue color), random coil (purple color), extended strand (red color), and beta turn (green color), of Elovl proteins in *E. tetradactylum* and *E. rhadinum*.

Predicting the protein structure is a fundamental prerequisite for understanding the function and possible interactions of a protein. In the present study, the secondary structures as well as three-dimensional structures of Elovl proteins in both *E. tetradactylum* and *E. rhadinum* were predicted using the SOPMA and Phyre2 programs, respectively. The protein structures of all the candidate Elovl proteins were modeled at > 90% confidence. The secondary structures of these proteins in *E. tetradactylum* revealed 40.86–50.30% alpha helixes, 28.10–28.10% random coil, 13.75–20.67% extended strand and 2.38–4.47% beta turn, while these ratios were predicted to be 47.55–53.27, 30.00–36.01, 6.99–18.12 and 2.38–4.75%, respectively, in *E. rhadinum* (Table 3). High ratio of alpha helixes and random coil in the Elovl protein structure might play important roles in fatty acids biosynthesis in fish, in accordance with the literature for the order Perciformes in *Perca fluviatilis*^36^. Additionally, the secondary structure pattern of Elovl proteins in the candidate *E. tetradactylum* and *E. rhadinum* species were highly similar (Fig. 3), indicating the probable similar biological functions as well as highly evolutionarily conserved Elovl genes in *Eleutheronema* species.

**Table 3:** Properties of the secondary structures of Elovl proteins.

Species	Protein	Alpha helix (%)	Beta turn (%)	Random coil (%)	Extended strand (%)
*E. tetradactylum*	*elovl1a*	46.65	3.83	30.99	18.53
	*elovl1b*	46.63	2.76	36.81	13.80
	*elovl4a*	43.02	4.47	31.84	20.67
	*elovl4b*	47.22	3.40	32.10	17.28
	*elovl5*	47.28	2.38	34.69	15.65
	*elovl6*	51.30	2.60	32.34	13.75
	*elovl6l*	50.36	3.65	28.10	17.88
	*elovl7a*	48.98	2.38	31.29	17.35
	*elovl8b*	40.86	4.12	40.69	14.33
*E. rhadinum*	*elovl1a*	50.16	2.56	31.31	15.97
	*elovl1b*	47.55	2.76	33.44	16.26
	*elovl4a*	48.32	4.75	32.40	14.53
	*elovl4b*	48.75	3.12	30.00	18.12
	*elovl5*	47.96	2.38	33.67	15.99
	*elovl6*	51.30	2.60	32.34	13.75
	*elovl6l*	48.54	3.28	31.02	17.15
	*elovl7a*	47.62	3.40	30.95	18.03
	*elovl8b*	53.27	3.72	36.01	6.99

**Figure 4:**
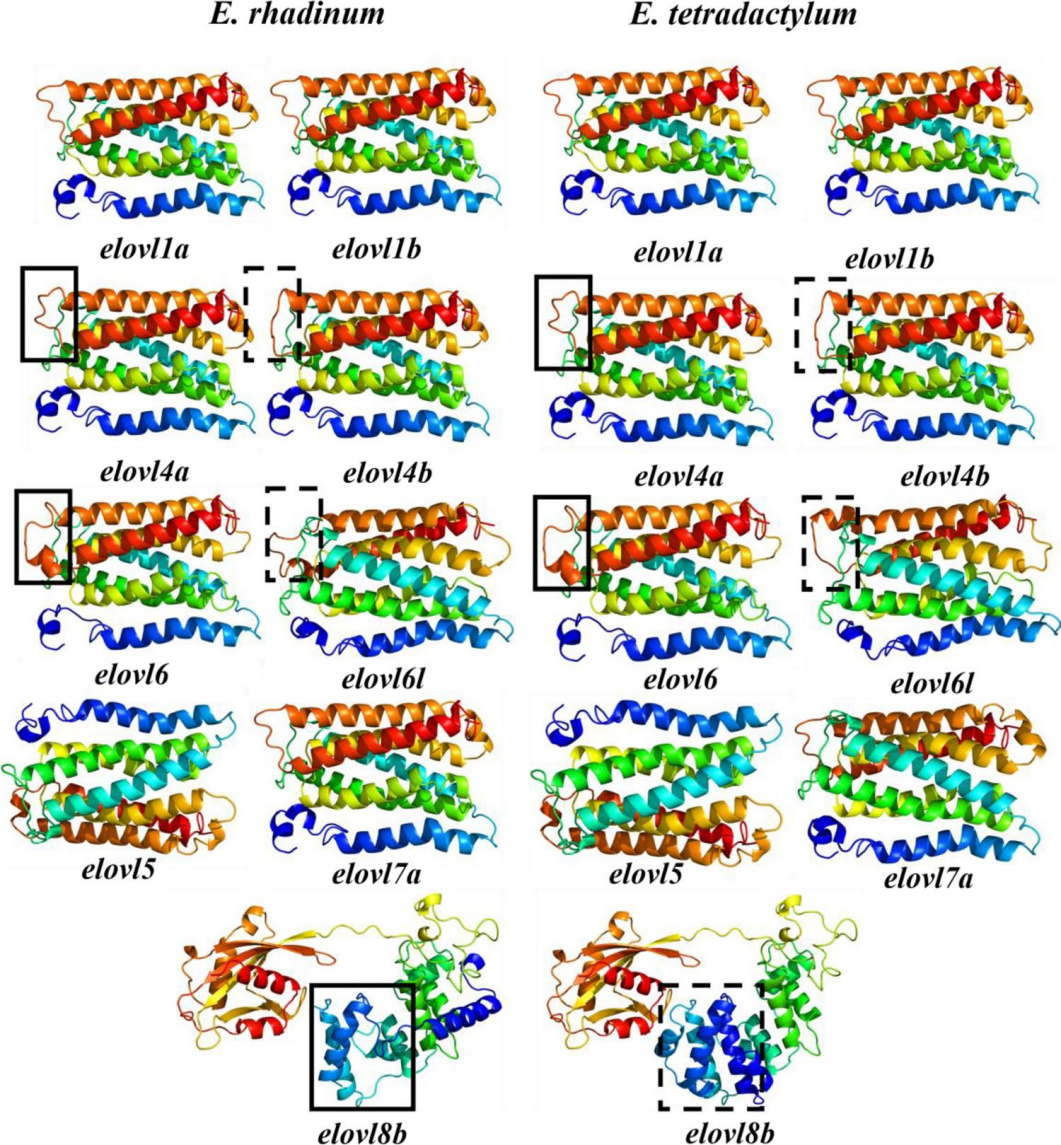
Three-dimensional modeling of Elovl proteins in *E. tetradactylum* and *E. rhadinum*. All models have confidence levels above 90%.

The 3D model results showed that all predicted Elovl proteins had complex 3D structures, composing of multiple secondary structures including alpha-helices, random coils, and others (Fig. 4). The Elovl proteins of different subfamilies showed different 3D configurations. The 3D structures of Elovl proteins also revealed the presence of the conserved domain in each Elovl protein, which showed a typical three-dimensional frame comprising of various parallel alpha-helixes. To assay the quality and accuracy of the predicted 3D model for the candidate Elovl proteins, the Ramachandran plot analysis was employed (Figure S1). In model validation, the qualities of the Elovl proteins model varied from 90 to 98% based on the Ramachandran plot analysis, suggesting the reasonably good quality and reliability of the predicted 3D models. These results indicated that the predicted 3D model of Elovl proteins could provide valuable information for the further comprehensive studies of molecular function in the fatty acids biosynthesis in *Eleutheronema* species. Additionally, the comparisons between these structures in *E. tetradactylum* and *E. rhadinum* suggested that the Elovl proteins encompassed the conserved structures. In addition, gene duplication resulted in obvious 3D structural variation in the duplicated genes, such as Elovl4 (*elovl4a* and *elovl4b*), Elovl6 (*elovl6* and *elovl6l*). The ascertained variations were revealed in duplicated Elovl proteins, and the diversities in these proteins structure may reflect their different obligations in the fatty acid biosynthesis and other biological processes.

**Figure 5:**
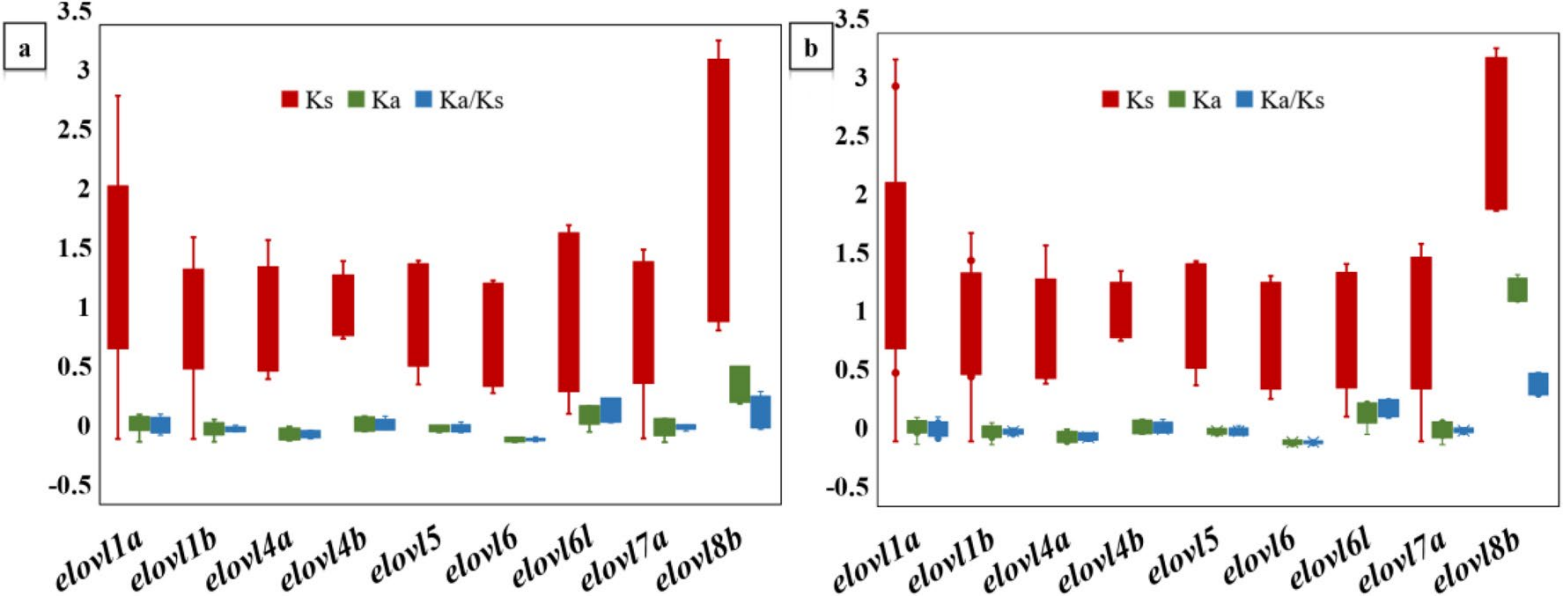
The evolutionary rates of the Elovl genes in (**a**) *E. tetradactylum* and (**b**) *E. rhadinum*. The Ka, Ks, and Ka/Ks values were demonstrated in boxplots with error lines.

To explore the functional selection pressures acting on Elovl gene family, Ka, Ks, and Ka/Ks ratios were calculated for each gene. Generally, Ka/Ks < 1 indicates purifying or negative selection, Ka/Ks = 1 represents neutral selection, and Ka/Ks > 1 indicates positive selection. In this study, we found that all the Ka/Ks ratios for each gene were less than 0.5, suggesting that they were subjected to strong purifying selection during evolution, and their functions might be evolutionarily conserved (Fig. 5). Therefore, theoretically, the Elovl genes in the *Eleutheronema* genus had eliminated deleterious mutations in the population through purification selection. Similar results were also observed in Elovl gene family of *Gymnocypris przewalskii* that no positive selection trace was detected in most members except *elovl2*^11^. Moreover, *elovl6l* and *elovl8b* showed a higher average Ka/Ks ratio than the other seven members, indicating that the evolution of *elovl6l* and *elovl8b* might be much less conservative and thereby could provide more variants for natural selection in *Eleutheronema* species.

### Chromosomal location, collinearity, and protein–protein interaction network analysis of Elovl genes

**Figure 6:**
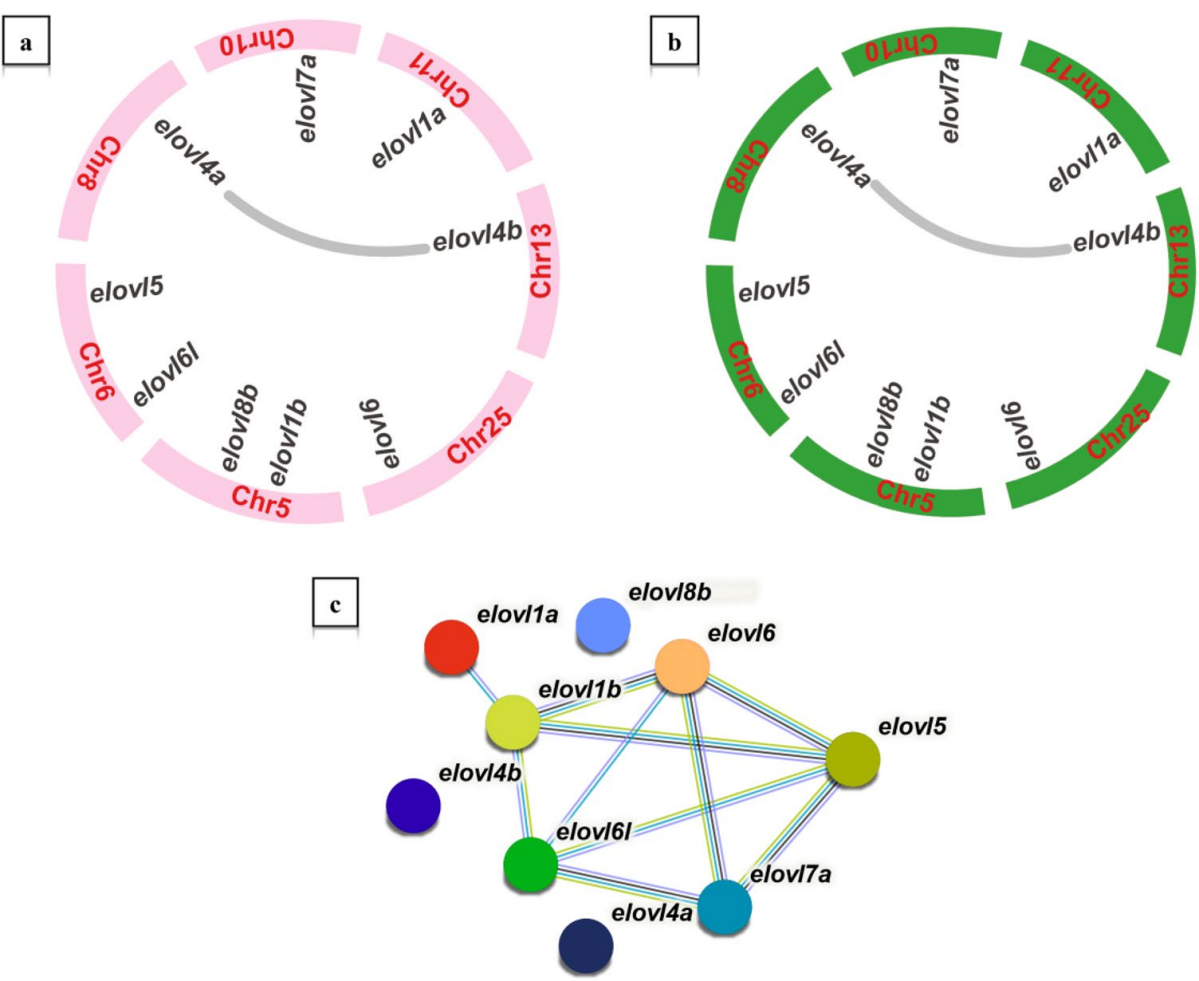
Chromosomal location and collinearity analysis of Elovl gene family in (**a**) *E. tetradactylum* and (**b**) *E. rhadinum*. Colored boxes represented chromosomes. Segmental duplication genes are connected with grey lines; (**c**) a protein–protein interaction network for Elovl genes based on their orthologs in zebrafish.

As shown in Fig. 6a and b, Elovl genes were randomly and unevenly distributed on seven chromosomes in both *E. tetradactylum* and *E. rhadinum*, including Chr5, Chr6, Chr8, Chr10, Chr11, Chr13, and Chr25. The Chr5 and Chr6 harbored two Elovl genes (*elovl1b* and *elovl8b* in Chr5, *elovl5* and *elovl6l* in Chr6), while other chromosomes each carried a single Elovl gene. Collinearity relationship analysis was performed to further investigate the gene duplication events within the Elovl gene family. The results revealed that a pair of segmental duplication genes (*elovl4a/4b*) showed collinear relationships. A chromosome-wide collinearity analysis also showed that the chromosomes were highly homologous between *E. tetradactylum* and *E. rhadinum*, including the Elovl gene family (Figure S2). To infer the protein interaction within Elovl gene family, we constructed the protein–protein interaction (PPI) network of the Elovl proteins based on the interaction relationship of the homologous Elovl proteins in zebrafish. The results showed that Elovl genes had close interaction with other members except for the *elovl4a/4b* and *elovl8b* (Fig. 6c), which suggested that they might participate in diverse functions by interacting with other proteins. Thus far, *elovl4a* and *elovl4b* were widely identified in most fish, which could effectively elongate PUFA substrates^37^. In addition, the *elovl4a/4b* were identified to be homologous proteins of zebrafish, indicating that the *elovl4* subtype was highly conserved during evolution and played important roles in the biosynthesis of LC-PUFA in *Eleutheronema*.

### Expression patterns of ELOVL genes in different tissues

**Figure 7:**
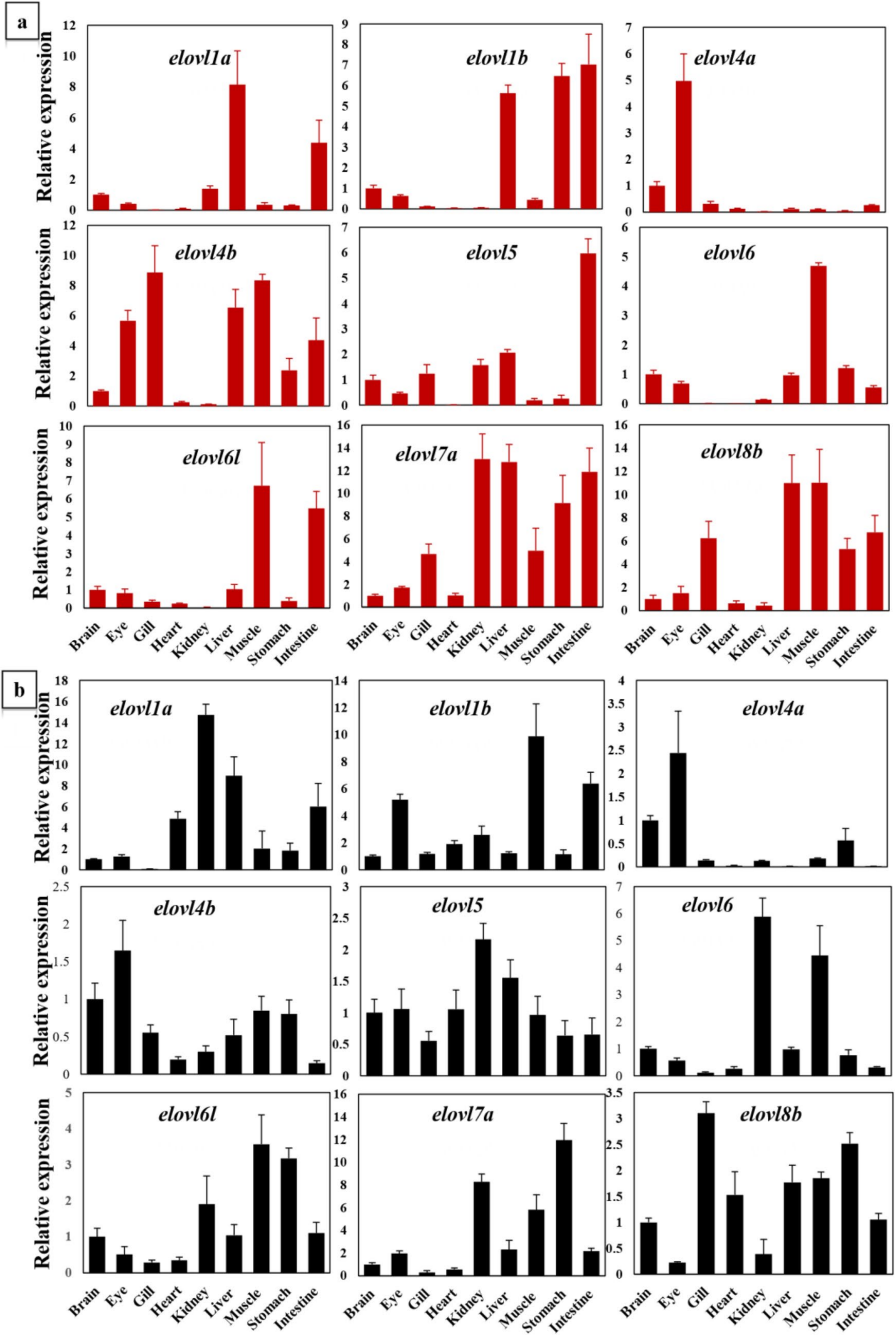
qPCR assessment of tissue distribution of *elovl1a, elovl1b, elovl4a, elovl4b, elovl5, elovl6, elovl6l, elovl7a*, and *elovl8b* gene expression in (**a**) *E. tetradactylum* and (**b**) *E. rhadinum* for various tissues including the brain, eye, gill, heart, kidney, liver, muscle, stomach, and intestine.

In the present study, we aimed to determine the expression patterns and gained insights into the potential functions of Elovl genes in the brain, eye, gill, heart, kidney, liver, muscle, stomach, and intestine. The expression patterns of Elovl genes in different tissues and species were distinct, suggesting the diverse roles during fish development (Fig. 7a and b). In our present study, the *elovl1a* and *elvovl1b* were expressed in a relatively narrow range of tissues, including the liver, stomach, and intestine. Some Elovl genes had much higher relative expression rates, e.g., elovl1a and elovl7a. The *elovl4a* was primarily distributed in the brain and eye, slightly expressed in gills while hardly detectable in other tissues, consistent with previous studies^37, 38^, which might play an important role in endogenous biosynthesis of LC-PUFA in the neural system of fish. In contrast to *elovl4a*, *elovl4b* was ubiquitously, instead of tissue-specific, expressed in most tissues while hardly examined in the heart and kidney. The *elovl4a* and *elovl4b* were two commonly paralogues in evolutionarily diverged fish species, and the striking difference in expression patterns between *elovl4a* and *elovl4b* might be due to the potential functional divergence of these two paralogues. In addition, *elovl8b*, the novel active member of the Elovl protein family, was expressed in several tissues, suggesting the essential roles in LC-PUFAs biosynthesis of teleost as indicated by a previous study^20^. Moreover, the differences in expression patterns among different Elovl genes indicated that these genes might possibly undergo functional divergence during evolution in the *Eleutheronema* genus. Overall, our present study firstly provided the preliminary organ-specific expression data of the Elovl gene family in *E. tetradactylum* and *E. rhadinum*, which could provide the foundation for further clarifying the function of these genes in the evolutionary development of the *Eleutheronema* genus.

## Conclusion

The Elovl gene family plays important roles in LC-PUFA biosynthesis in fish, thus the identification of the Elovl gene family in *Eleutheronema* species is essential to understand its underlying molecular basis of fatty acids biosynthesis. In this study, we first identified 9 Elovl genes from two *Eleutheronema* fish, including *E. tetradactylum* and *E. rhadinum* genome. We found that the Elovl gene family could be classified into six subfamilies in *Eleutheronema* species, including *elovl1a/1b*, *elovl4a/4b*, *elovl5*, *elovl6/6 l*, *elovl7a*, and *elovl8b*, which were consistently supported by their gene and protein structures and conserved motifs. The gene loss of *elovl2*, *elovl3*, *elovl7b,* and *elovl8a* was observed in *Eleutheronema* genus. All found genes showed highly conserved gene and protein structures and motif composition except for elovl8b. We also found that the Ka/Ks ratios for each gene were less than 0.5, suggesting that the Elovl genes in the *Eleutheronema* genus were subjected to strong purifying selection during evolution. The expression patterns of Elovl genes across tissues and species were distinct, suggesting possibly functional divergence during evolution in the *Eleutheronema* genus. Overall, our present study firstly presented comprehensive information about the sequence characteristics, phylogenetic relationships, chromosome distribution, selection pressure, and expression patterns of Elovl gene in the *Eleutheronema* genus. The results could provide the basis for further research on the function of the Elovl gene family in marine fish, thus facilitating the molecular breeding of fatty acids-enriched fishes with potential benefits to farmers and the health of consumers.

## Supplementary Information

Supplementary Information.

## Data Availability

The sequence data obtained in this study have been deposited at GenBank of NCBI at OP391488-OP391504 (https://www.ncbi.nlm.nih.gov/).
