# Supplementary material for: Genome-wide identification and expression profile of Elovl genes in threadfin fish Eleutheronema
Source: Sci Rep. 2023 Jan 19;13:1080. doi: 10.1038/s41598-023-28342-4 (PMC9852283; doi:10.1038/s41598-023-28342-4)
Supplement: Supplementary file 1 — Supplementary Information. [file 41598_2023_28342_MOESM1_ESM.docx]

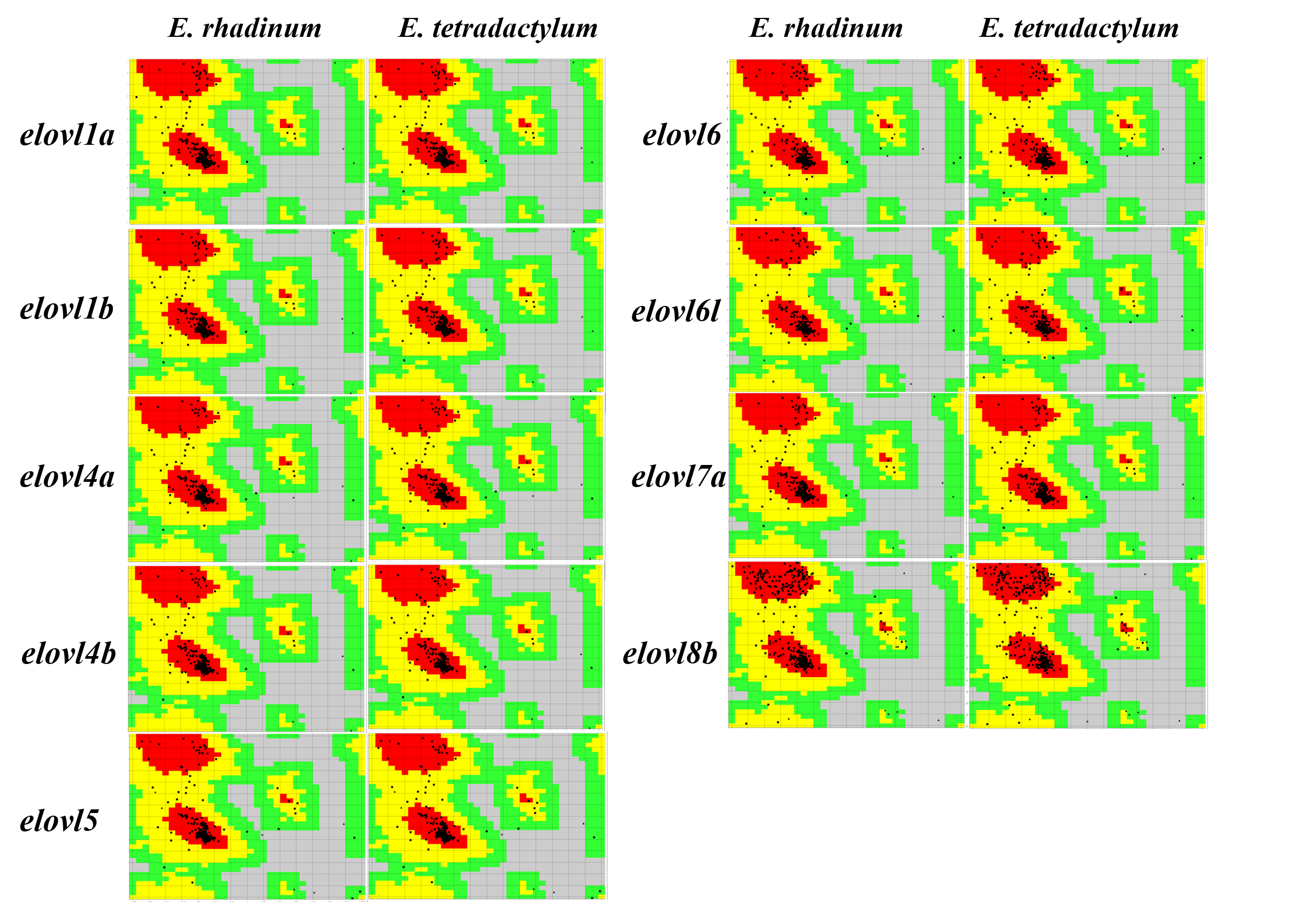


**Figure S1**. Validation of the predicted models through Ramachandran plot analysis in *E. tetradactylum* and *E. rhadinum*.


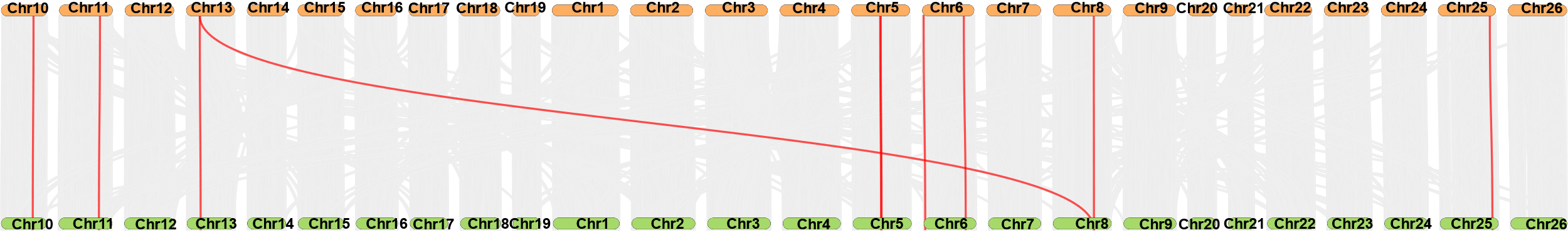


**Figure S2**. Chromosome-wide collinearity analysis between *E. tetradactylum* and *E. rhadinum*. Analysis of syntenic Elovl genes between *E. tetradactylum* (up) and *E. rhadinum* (down). The collinear blocks between *E. tetradactylum* and *E. rhadinum* genomes are indicated using grey background lines, whereas the red lines highlight the syntenic Elovl gene pairs.

**Table S1. Conserved motif sequences of Elovl genes in *E. tetradactylum* and *E. rhadinum***

| **motif** | **Protein sequences** |
| --- | --- |
| motif1 | RKKNNQITFLHVYHHSTMLLLWWYGIKWV |
| motif2 | PKMQKYLWWKKYLTAIQLIQFVLVIGHTSQYLFM |
| motif3 | GPRYMKNRKPFDLRKALIVYNLSLVVLSF |
| motif4 | FHATMNSFVHVIMYSYYGLAA |
| motif5 | RMASVLWWYYFSKGIELLDTVFFI |
| motif6 | YIVYELLMAGWATGYSYRCDPVDYSBSPQ |
| motif7 | YGVTFIILFSNFYYQAYIKGK |
| motif8 | SDAVEFYDYLLTIADPRVEBWPLMDNPVPTTAILLAYLLFV |
| motif9 | HILSSDGFKRSICDQSFYTGPVNKFWAYAFVLSKAPELGDT |
| motif10 | ITLNQITQVANGNVVSGLTEEWMQEDDCGSSKV |
